# Cardiac myxoma discovery behind chronic carotid occlusion in stroke

**DOI:** 10.21542/gcsp.2024.49

**Published:** 2024-12-31

**Authors:** Ibrahim Oumarou Hamissou, Jerome Wintzer-Wehekind

**Affiliations:** 1 Department of Interventional Cardiology, Emile Roux Health Care Center, Le Puy-en-Velay, France; 2 Department of Cardiology, University Mohammed-V of Rabat, Morocco

## Abstract

A 57-year-old female smoker presented with a left-sided ischemic stroke, leading to the discovery of two concurrent potential embolic sources: a chronic left internal carotid artery occlusion and a left atrial myxoma. While cardiac myxomas are known sources of cerebral emboli, this case presented a unique management challenge due to the coexisting carotid pathology. Despite successful surgical resection of the myxoma, the patient experienced a subsequent stroke one month postoperatively. This case highlights the complex clinical decision-making required when managing patients with multiple potential sources of cerebral embolism.

## Introduction

Myxomas are benign cardiac tumors that predominantly occur in the left atrium. They are often discovered incidentally during imaging studies performed for unrelated conditions, or during the workup of complications such as stroke. When stroke occurs, determining its exact etiology requires comprehensive investigation, particularly challenging in cases with multiple potential sources like concurrent myxoma and carotid occlusion, as demonstrated in our patient. This report presents the clinical features, diagnostic approach, and therapeutic management of such a complex case.

## History

The patient was admitted on the 6th August 2023 for a right-sided hemifacial paresthesia. She had been smoking for the past fifteen years, with no other past medical history. She presetned with no cardiovascular or respiratory symptoms and her blood pressure was 123/83 mmHg. Her heart sounds were normal without murmurs. There were no clinical signs of heart failure, however, the left carotid pulse was absent on palpation.

## Differential diagnosis

Thrombus and malignant tumors of the left atrium are the main differential diagnoses.

**Figure 1.**
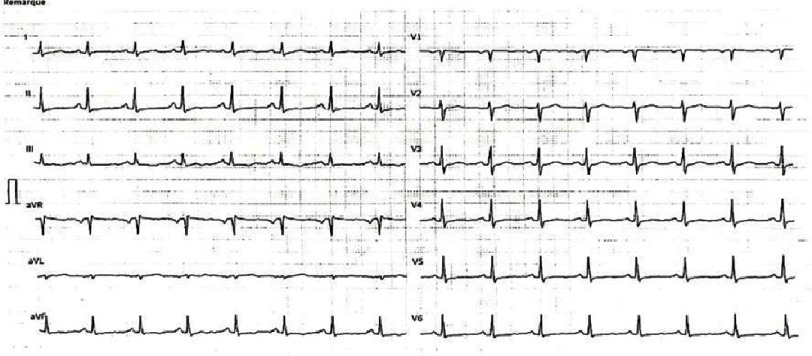
Twelve-lead electrocardiogram showing a sinus rhythm.

**Figure 2.**
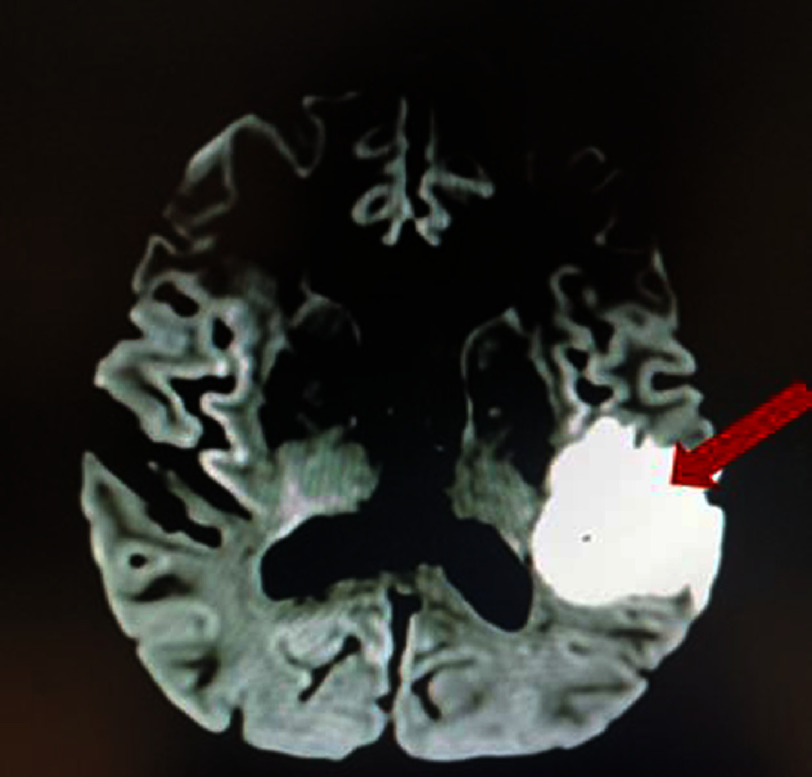
Cerebral angio-MRI showing a large stroke in the left Sylvian territory on diffusion sequence.

**Figure 3.**
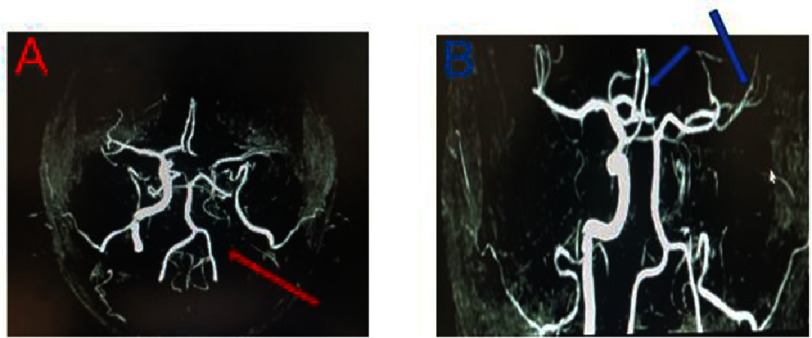
Angio-MRI of the supra-aortic trunks showing an absence of flow at the ostium of the left internal carotid artery (A: red arrow) associated with a collateral flow from communicating arteries (B: blue arrows) resulting first in a complete and chronic occlusion.

## Investigations

The investigation started the same day with an electrocardiogram showing a sinus rhythm (Figure 1), without any disorder. Brain angio-MRI found on the diffusion sequence, a hypersignal (arrow) of the left superficial Sylvian territory, suggesting an acute large ischemic lesion (Figure 2). The angio-MRI of the supra-aortic trunks at the same time showed an absence of flow at the left internal carotid artery (red arrow) associated with a normal flow at its terminal portion from communicating arteries (star) (Figure 3).

**Figure 4.**
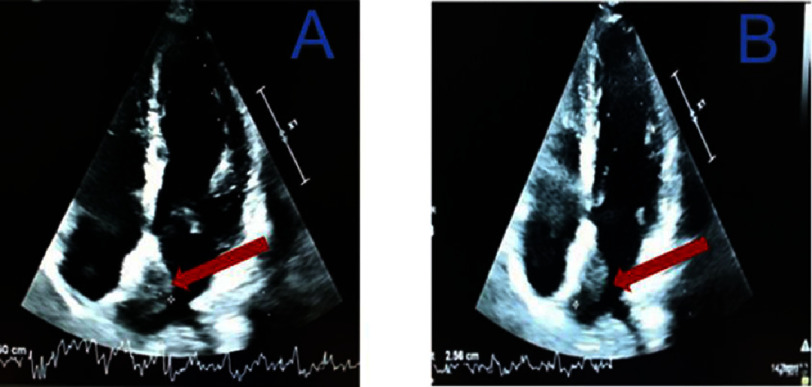
Transthoracic echocardiogram showing a left intra-atrial mass (arrow) attached to the inter-atrial septum and measuring 2.5 cm (A = horizontal diameter and B = vertical diameter).

The chronic carotid lesion could be mistaken for the cause of the stroke. Transthoracic echocardiography revealed a 2.5 cm mass in the left atrium attached to the inter-atrial septum, as visualized in apical four-chamber view (Figure 4).

**Figure 5.**
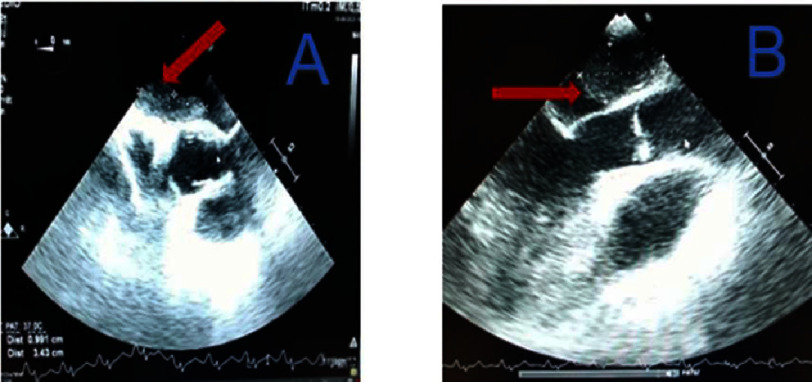
Transesophageal echocardiography revealing a myxoma of the left atrium (arrow) measuring 3.43 cm (A) and 4.5 cm in large diameter (B) in the left ventricular outflow tract.

The cardiac cavities were of normal size and the left ventricular ejection fraction was 65%. A transesophageal echocardiogram performed two days later (8th August 2023) confirmed the presence of a myxoma of the left atrium, measuring 4.5 cm at its largest diameter in the left ventricular outflow tract view (Figure 5). There was no atrial thrombus, nor a patent foramen oval during saline contrast study; and the native cardiac valves were thin and flexible. Continuous cardiac telemetry monitoring over 48 h showed no evidence of atrial fibrillation or other arrhythmias associated with embolic risk. All blood tests were normal.

**Figure 6.**
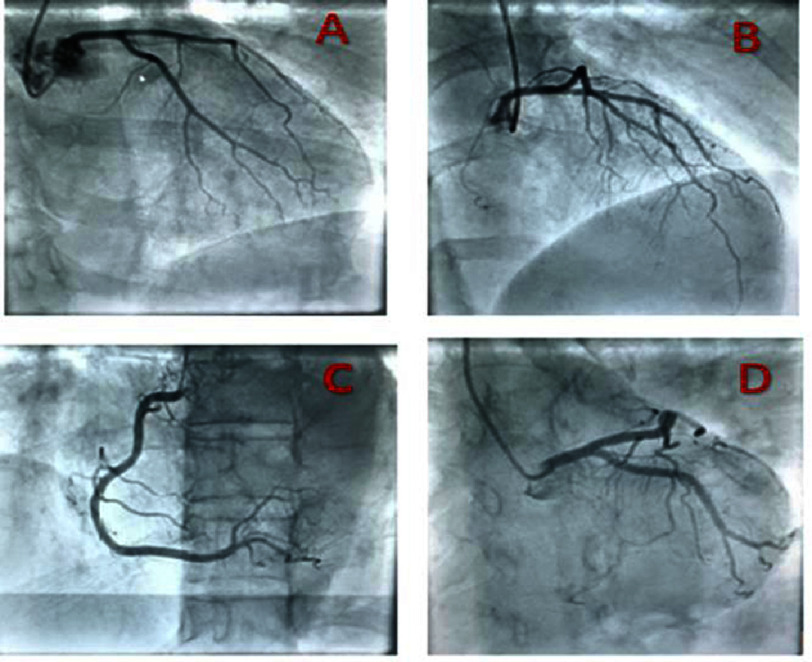
(A) Caudal right anterior oblique view showing the circumflex-marginal arteries which are normal, (B) Cranial right anterior oblique view showing the left anterior descending artery (normal), (C) View showing the right coronary artery which is normal, (D) Caudal left anterior oblique view showing the main left coronary artery which is normal.

**Figure 7.**
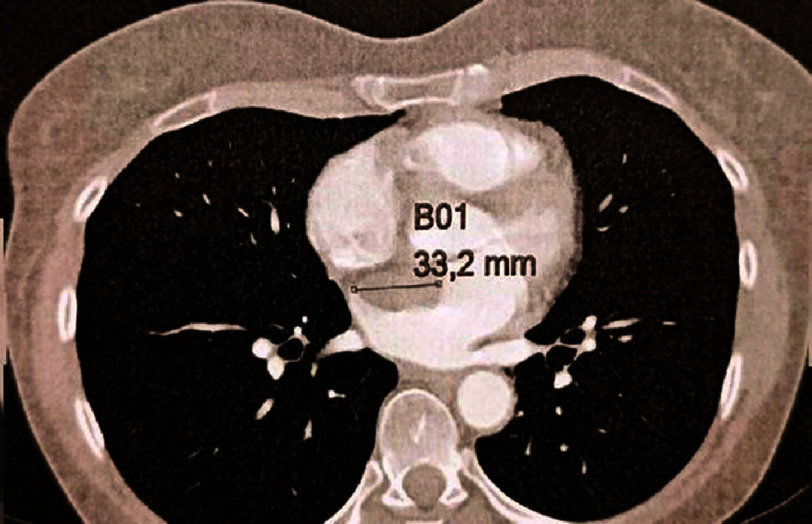
Thoraco-abdomino-pelvic contrast tomography scan showing the myxoma of the left atrium measuring 3.32 cm.

## Management

There was a surgical indication to remove the myxoma, and further explorations were necessary as part of the preoperative assessment. A coronary angiogram on 11th August 2023 indicated normal coronary arteries (Figure 6). On 15th August 2023, a thoraco-abdominopelvic contrast tomography scan confirmed the previously identified left atrial myxoma, measuring 3.3 cm, with no other abnormal findings (Figure 7). The patient was transferred to the cardiac surgery department that same day.

**Figure 8.**
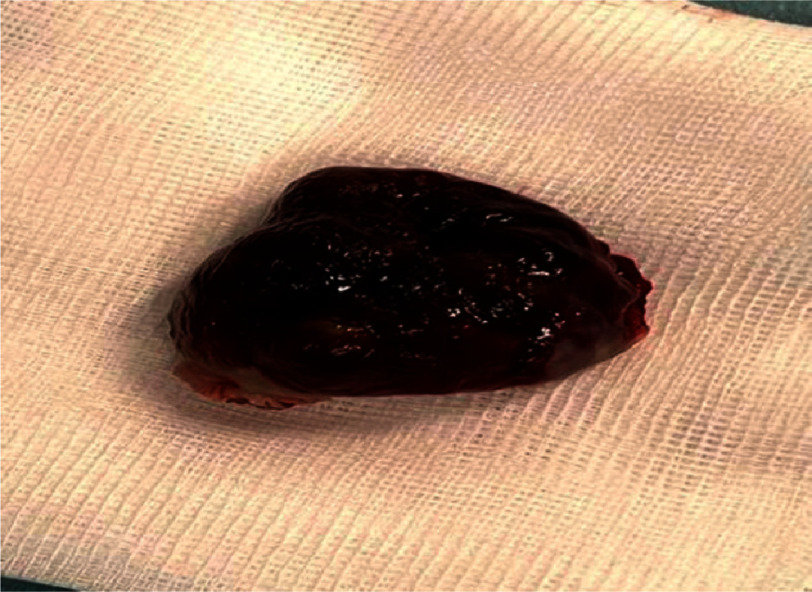
Macroscopic aspect of the polylobulated myxoma.

The surgical procedure, which consisted of complete excision of the myxoma associated with the inter-atrial septum reparation with a Dacron patch, was completed 2 days later. The excised myxoma was found to be friable and polylobulated in nature (Figure 8). The patient was discharged four days later (21st August 2023) continuing her previous regimen of antiplatelet therapy and statins.

One month after surgery she presented with a new onset stroke. The electrocardiogram showed a sinus rhythm, but transesophageal echocardiogram showed no intracardiac mass. The saline contrast test was negative. Her CHA2DS2VA score was at 3 and we finally introduced in our patient a long-term anticoagulant treatment based on Apixaban 5mg twice daily, instead of the antiplatelet therapy.

## Discussion

Myxoma represents 50% of all benign cardiac tumors and in 75% of cases occurs in the left atrium^1^. It is discovered incidentally during chest imaging or during its complication^2^. The myxoma appears as a polyploid mass on echocardiogram, very mobile, attached by its pedicle to the inter atrial septum in the region of the fossa ovalis. As for the thrombus, the main differential diagnosis, it has the appearance of a homogeneous mass, most often in a context of mitral stenosis or atrial fibrillation^3^. Contrast tomography scan or magnetic resonance imaging and coronary angiogram are often requested as part of the preoperative assessment. Histological and genetic tests are rarely performed^3^. Furthermore, supra-aortic imaging and rhythmic explorations are useful to look for other thromboembolic etiologies. The most frequent complications are embolisms in 40% of cases, follwed by atrioventricular obstruction, and myxoma infection^3^. Surgical intervention usually consists of complete excision of the tumor mass^4^. Post-operative complications can include the recurrence of the myxoma, an atrial fibrillation, or rarely, the formation of thrombus at the excision site.

In our patient, the etiology of her stroke was related to the myxoma found in the left atrium due to the large aspect of the cerebral ischemia, the location of the myxoma, its mobility, as well as its multilobulated and friable nature. In addition, the absence of other thromboembolic causes supports this diagnosis. Left internal carotid artery occlusion did not explain the symptoms and the presence of normal downstream vascularization showed that it was likely a chronic lesion.

The presence of this lesion could be misleading, acting as the proverbial “tree” that obscures the “forest” - with the carotid occlusion being the tree that masks the true underlying cause (the forest) - in this case the myxoma.

The recurrence of our patient’s stroke was related to a postoperative atrial fibrillation. This post-operative, paroxysmal atrial fibrillation often occurs in the week following cardiac surgery and 50% of patients return to a sinus rhythm in less than 24 h^5,6^.

After evaluating our patient’s hemorrhagic and embolic risk profile, with a CHA2DS2VA score of 3 indicating elevated embolic risk, we initiated anticoagulation with apixaban 5 mg twice daily for a minimum duration of one month.

The rationale for oral anticoagulation in postoperative atrial fibrillation is supported by observational studies demonstrating significantly higher stroke risk in these patients. Furthermore, new-onset postoperative atrial fibrillation often predicts future arrhythmia development, with one observational study showing that 50% of patients eventually developed chronic atrial fibrillation, including 18% within the first year^7^.

## Conclusion

Myxoma has been shown to responsible for 15.3% of cases of stroke within certain populations. The coexistence of a myxoma and significant carotid atherosclerotic disease can complicate stroke etiology determination. However, myxoma-related infarcts tend to be multifocal and extensive, whereas carotid-origin strokes typically follow well-defined territorial patterns and vascular distributions.

Post-operative atrial fibrillation is common with cardiac surgery and anticoagulation for at least one month should be considered, especially in high thrombo-embolic risk patients.

## Follow-up

Our patient improved clinically with all vital signs within normal limits. The electrocardiogram shows a sinus rhythm and blood tests were unremarkable. Echocardiogram showed a normal left ventricular ejection fraction at 63%, a little septal dyskinesia with minimal epicardial effusion. She was discharged with a close cardiac follow-up.
